# Analyzing Factors Associated with the Behavior-Change Stage of Colorectal Cancer Screening

**DOI:** 10.3390/healthcare10081492

**Published:** 2022-08-08

**Authors:** Jisun Lee

**Affiliations:** Department of Nursing, Honam University, Gwangju 62399, Korea; 2018091@honam.ac.kr

**Keywords:** colorectal neoplasm, screening, health behavior, barriers, self-efficacy

## Abstract

This study attempted to identify the stages of colorectal cancer screening (CRCS) behavior change by applying the precaution adoption process model (PAPM) and to examine the factors affecting each stage. A quantitative, descriptive, cross-sectional design was used. A total of 305 workers at one of the largest industrial complexes in South Korea were allocated using convenience sampling. Data were analyzed using independent *t*-test, one way ANOVA and multi-nominal logistic regression using SPSS 25.0 (SPSS Inc., Chicago, IL, USA). Most of the subjects were in the undecided-to-act stage. The factors affecting each stage were gender, marital status, family history, private insurance subscription, perceived barrier, and self-efficacy. It is critical to select a target group considering the behavioral change stage to establish a strategy for improving the CRCS rate. Developing and implementing a CRCS education program in consideration of the behavioral change stage will be a strategy to increase the examination of effectiveness of CRCS.

## 1. Introduction

The worldwide incidence rate of colorectal cancer (CRC) is the third highest after lung cancer and breast cancer, and its mortality rate is the second highest after lung cancer [1]. In 2018, the incidence rate of CRC in South Korea was 44.5 per 100,000 people, the second highest in the world after Hungary, which recorded 51.2 cases [2].

The World Health Organization emphasized early screening as a preventive measure to reduce the occurrence of or death from cancer and urged national interest and efforts. In South Korea, free cancer screenings have been made available for certain types of cancers as a policy since 1999, and the screening subjects and items have been expanded gradually to include CRC since 2004. In 2020, the screening rate for the five major cancers (gastric cancer, CRC, liver cancer, breast cancer, and cervical cancer) was 49.2%, an increase from 29.7% in 2017; however, the CRC screening rate was 36.9%, which remains lower than the overall screening rate [3]. In South Korea, the fecal occult blood test is conducted as a first-level test every year for 50-year-old adults, and if the test result is positive, double-contrast radiography or a colonoscopy is conducted as a second-level test [4]. Most CRC cases start as an adenomatous polyp and progress to CRC over a long period of time, without apparent symptoms until the terminal stage [5]. Due to this nature of CRC, the early detection and removal of adenoma through cancer screening can prevent the development of cancer, thereby lowering the incidence of CRC and improving survival rates [6]. Despite the effectiveness of colorectal cancer screening (CRCS), the screening rate of CRC is significantly lower than that of other cancer types, which makes it necessary to analyze why the rate is low and to prepare countermeasures. Previous studies reported that high self-efficacy, low perceived barrier, family history of cancer, and the screening experience of the spouse have a positive influence on CRCS [7], while low educational level, income, and single status are negative influencing factors on CRCS [8]. Although the importance of CRCS is being emphasized, the CRCS rate has not improved; thus, a more systematic study is needed to improve it. Therefore, for CRCS behavior change, it is necessary to accurately identify the stage of change in the subject’s behavior, identify the factors affecting the CRCS behavior change, and implement customized intervention.

When people choose healthy behaviors, they deliberately select a behavior to adopt new precautions or stop risky behaviors [9]. The stages in which an individual recognizes the need for healthy behavior and takes action occur through a series of processes [10]. The precaution adoption process model (PAPM), a beneficial model for explaining health behavior changes, explains the stages of health behaviors derived from the social learning theory and the health belief model by focusing on individual psychological characteristics [11,12]. In particular, PAPM focuses on an individual’s psychological process, such as subjective thoughts and beliefs, and behavioral changes and explains how to implement such a decision.

PAPM has seven stages that are classified ranging from lack of awareness to action—stage 1: unaware of the issue; stage 2: aware of but unengaged with the issue; stage 3: undecided about acting; stage 4: decided not to act; stage 5: decided to act but not yet acting; stage 6: acting; and stage 7: maintenance [11,12]. PAPM subdivides the “pre-contemplation stage” in the trans-theoretical model, also known as the stages of change theory, into stages 1–3 in PAPM; thus, it is a beneficial model when behavior-change awareness is low or for identifying behavior changes in relation to health risk [13]. Meanwhile, most studies on CRCS viewed the practice of health behavior as a dichotomous category of phenomena, and they mainly examined the behavior-related factors as a single predictive formula such as attitude, ability, and knowledge.

Regarding this, in this study, PAPM is applied to identify the stages of CRCS behavior change to identify the factors affecting each one and use them as core data for customized intervention strategies for each stage of CRCS behavior change. The specific research objectives are as follows: 

First, the demographic characteristics according to the CRCS behavior-change stages are identified.

Second, the difference in health beliefs and self-efficacy according to the CRCS behavior-change stages is identified.

Third, the influencing factors for each CRCS behavior-change stage are identified.

## 2. Materials and Methods

### 2.1. Research Design

A quantitative, descriptive, cross-sectional design was employed. 

### 2.2. Study Participants

The subject of this study is a convenience sample of workers at one of the largest industrial complexes in South Korea. The subjects of this study were workers over the age of 50 who understood its purpose and agreed to participate [14]. Subjects with a history of or currently undergoing treatment for any cancer were excluded due to the different screening criteria for the high-risk CRC group.

The sample size of this research was based on the previous study [15], and for multinomial logistic regression analysis using the G*Power program, significance level (α) = 0.05, power (1 − β) = 0.80, odds ratio = 1.5, and probability Ho = 0.5, 208 people were calculated. The survey was conducted for a total of 310 people, considering the dropout rate, to secure the number of subjects for each stage of CRCS behavior change. A total of 305 responses (the response rate was 98.4%) was used for the final analysis, excluding 5 copies with missing or incorrect responses among the recovered questionnaires. 

### 2.3. Research Instruments

#### 2.3.1. CRCS Behavior-Change Stage

**Figure 1:**
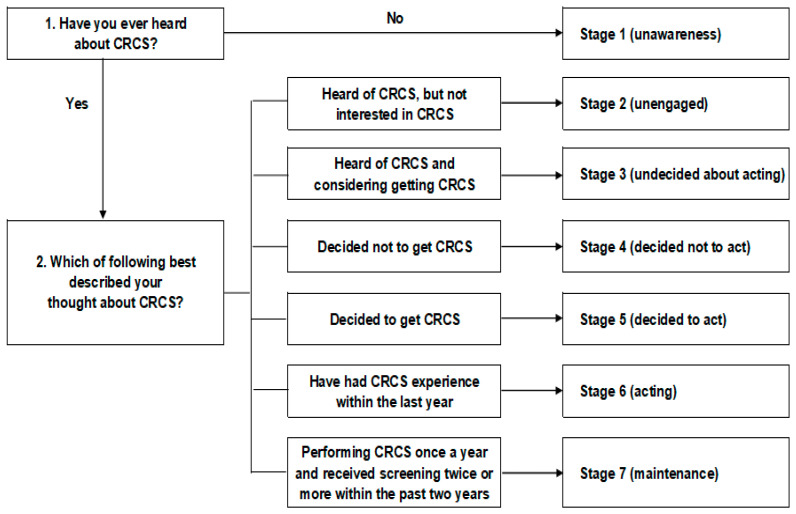
Stages of CRCS behavior change.

The CRCS behavior-change stages were classified based on the seven stages of PAPM. In the flow chart, the higher the level is, the more the CRCS behavior was maintained, and the participants were asked to mark the level corresponding to their status. Each one was based on the perception, intention to act, and experience of CRCS, and the detailed classification of each stage is as follows (Figure 1):

Stage 1 (unawareness): Never heard of CRCS;

Stage 2 (unengaged): Heard of CRCS, but not interested in CRCS;

Stage 3 (undecided about acting): Heard of CRCS and considering getting CRCS;

Stage 4 (decided not to act): Decided not to get CRCS;

Stage 5 (decided to act): Decided to get CRCS;

Stage 6 (acting): Have had a CRCS experience within the last year;

Stage 7 (maintenance): Performing CRCS once a year and received screening twice or more within the past two years.

#### 2.3.2. Health Belief

The Champion health belief model scale (CHBMS) developed by Champion [16] for breast cancer patients was used to assess health beliefs. We modified the scale by replacing breast cancer with CRC and used it after obtaining author approval. This scale consists of five items of perceived susceptibility, seven items of perceived severity, six items of perceived benefit, and six items of perceived barrier on a 5-point Likert scale, with higher values indicating high health belief. However, higher perceived barrier scores indicate lower perceived barriers. The Cronbach’s alpha was 0.60–0.78 when the scale was developed, and in this study, Cronbach’s alpha was 0.76 for perceived susceptibility, 0.90 for perceived severity, 0.80 for perceived benefit, and 0.67 for the perceived barrier. 

#### 2.3.3. Self-Efficacy

For self-efficacy, five items on perceived confidence from CHBMS developed by Champion [16] were modified and used with author approval. Each item is on a 5-point Likert scale, with higher values indicating higher self-efficacy. Cronbach’s alpha was 0.88 at the time of tool development and was 0.87 in this study. 

#### 2.3.4. General Characteristics

The demographic characteristics were investigated by way of age, gender, education, marital status, employment status, family history, and private insurance. 

### 2.4. Data Collection and Ethical Consideration

This study was approved by the Institutional Review Board of Honam University (Approval number: 1041223-202008-HR-14). The data collection period was from August to December 2020, and data were collected using a self-reporting questionnaire. First, cooperation from the research target institution was requested, and approval was obtained according to each institution’s procedures. Subjects selected by the relevant institution read the research description and consent form and participated in the survey following their voluntarily consent. Subjects who participated in the survey received a gift in return, considering the average response time of 10 min. The informed consent form consisted of information on the purpose and content of the study, subject rights, anonymity, confidentiality, voluntary consent with the possibility to withdraw, and a guarantee that the collected data were for research purposes alone. 

### 2.5. Data Analyses

Statistical analysis was performed using SPSS 25.0 for Windows (IBM Corp, Armonk, NY, USA), and all *p*-values were two-sided with a significance level set at 5%. 

(1) Descriptive statistics, independent *t*-test, and one-way ANOVA were used to analyze demographic characteristics according to the CRCS behavior-change stage; (2) one-way ANOVA was used for differences in health belief and self-efficacy according to the CRCS behavior-change stage; and (3) multi-nominal logistic regression was used for influencing factors for each stage of CRCS behavior change. Since the basic assumption of multivariate analysis should be a normal distribution, skewness and kurtosis were evaluated to confirm that the conditions of the normal distribution were satisfied.

## 3. Results

### 3.1. Demographic Characteristics According to CRCS Behavior-Change Stages

The average age of the subjects of this study was 53.2 years; there were 151 males (49.5%) and 154 females (50.5%). Regarding the CRCS behavior-change stage distribution, among the participants, 121 people (39.7%) corresponded to stage 3 (undecided about acting), and none corresponded to stage 4 (decided not to act). 

Regarding the characteristics of subjects in stage 7, 42 (27.8%) were male, more than 25 (16.2%) were female, 63 (22.7%) were married, and more than 4 (14.3%) were not married. There were 61 (24.75) people with private insurance and more than 6 (10.3%) without it (Table 1). 

### 3.2. Dfferences in Health Beliefs and Self-Efficacy According to CRCS Behavior-Change Stages

In terms of health beliefs, according to the CRCS behavior-change stage, the perceived barrier was highest with 3.29 points at stage 1 (unawareness), tending to decrease toward stage 7 (maintenance) with 2.78 points, and the difference was statistically significant (F = 3.62, *p* = 0.003). 

Self-efficacy was the highest with 3.97 points in stage 6 (acting) and lowest with 3.45 points in stage 1 (unawareness). Post hoc analysis showed that the self-efficacy in stage 1 (unawareness) was significantly lower than in stage 5 (decided to act) and stage 6 (acting) (F = 6.16, *p* < 0.001) (Table 2). 

### 3.3. Influencing Factors for Each Stage of CRCS Behavior Change

Regarding the predictors of the CRCS behavior-change stage, multinomial logistic regression analysis was performed using statistically significant variables as independent variables based on the results of cross-analysis and variance analysis. 

The independent variables included in the analysis were age, gender, marital status, family history, private insurance, perceived barrier, and self-efficacy. Prior to analysis, nominal variables such as gender, marital status, family history, and private insurance were converted into dummy variables to fit the regression analysis. The standard category of the dependent variable was set as “maintenance stage”.

As a result, the influencing factors of the CRCS behavior-change stage were gender, marital status, family history, private insurance, perceived barriers, and self-efficacy (Table 3). 

In terms of gender, the odds that men belonged to stage 3 (undecided about acting), stage 5 (decided to act), and stage 6 (acting) were higher by 0.22 times, 0.04 times, and 0.23 times, respectively, compared to stage 7 (maintenance stage). 

For marital status, the odds were 0.33 times higher for being in stage 3 (undecided about acting) than stage 7 (maintenance) when subjects had a spouse. 

For family history, we found that the odds of belonging to stage 6 (acting) increased by 6.37 times compared to stage 7 (maintenance) when subjects had a family history of CRC. 

In the case of private insurance, we found that the odds of being in stage 3 (undecided about acting) increased by 0.29 times compared to stage 7 (maintenance) when subjects had private insurance. 

For perceived barrier, the odds of belonging to stage 1 (unawareness), stage 2 (unengaged), and stage 3 (undecided about acting) increased by 4.10 times, 3.25 times, and 1.84 times, respectively, compared to stage 7 (maintenance) when the scores were high.

In terms of self-efficacy, an increase of 1 point increased the odds of belonging to stage 6 (acting) by 0.22 times compared to stage 7 (maintenance).

## 4. Discussion

This study attempted to identify the stages of CRCS behavior change by applying PAPM and to examine the factors affecting each stage. The results of examining the stages of CRCS behavior change suggested that the majority of subjects were in the undecided-about-acting stage, followed by the maintenance stage, acting stage, decided-to-act stage, unengaged stage, and unawareness stage. These results were consistent with the results of a PAPM-based study on the distribution of CRCS behavior changes targeting 486 adults 50 years of age or older in 2010 [17]. 

Meanwhile, in the study examining breast cancer screening behavior, the most common behavioral decision stage was the decided-to-act stage, followed by the unengaged stage and the maintenance stage [18]. In another study analyzing breast cancer screening behavior, the most frequent behavior decision stage was also the decided-to-act stage, followed by the undecided-about-acting stage and the unengaged stage [18]. As such, while there is a difference in the distribution for each stage of behavior change depending on the type of examination, most of the target groups belong to the undecided-about-acting stage or the decided-to-act stage. To improve the CRCS rate through the distribution of behavior-change stages, it is necessary to develop an intensive intervention strategy so that subjects in the undecided-about-acting stage can move forward and decide their course of action.

The total percentage of subjects who underwent CRCS in this study was 40.4% (18.4% in the acting stage and 22% in the maintenance stage), higher than the overall screening rate of CRC in South Korea, which was 36.9%. It is assumed to be higher than the national cancer screening rate because the subjects of this study were industrial complex workers and participated in screenings as part of the occupational health service. The leading reason for not having a CRCS done was lack of knowledge about screening behavior [14], which has been shown to lead to incorrect perception and judgment of the subject and affect examination [7]. Therefore, to improve the CRCS rate, efforts are required to assess the knowledge level of individuals and provide customized information according to individual characteristics and needs.

The CRCS rate (in the acting and maintenance stages) was about 20% higher in males than in females, and this was consistent with the CRCS behavior survey results of the National Cancer Center [14]. The difference occurred because males are relatively more active in social activities than females and have more opportunities to access Korean health resources. Considering that the subjects of this study were industrial workers, and the gender ratio was almost the same, it can be predicted that other factors, such as the type of work and length of service, influenced the behavior-change stage. Therefore, it will be necessary for future studies to identify factors that influence gender differences in screening rates.

For subjects with spouses, the number of people in the acting stage and maintenance stage was the highest and lowest in the unawareness stage. This was consistent with the results of a study that reported that subjects with a spouse had a higher screening rate than subjects without a spouse [7,19]. In particular, this supports the previous study that reported that a spouse has a significant influence on CRCS health behavior decisions and that the screening probability increased when the spouse frequently recommended cancer screening [20]. In an environment where a social context is shared, such as in a marriage, it can be predicted that interpersonal attitudes or surrounding environments significantly influence decision making. Meanwhile, for the maintenance stage, significantly more subjects were distributed in the undecided-to-act stage when they had a spouse compared to when they had no spouse. Therefore, for those who are married and are undecided to act, it will be effective to intervene in focusing on social support and recommendation through the spouse.

When examining the stage of behavior change according to family history in the context of family interaction, the CRCS rate (acting stage and maintenance stage) was similar for subjects with and without family history, but the distribution of subjects in the unawareness stage was 1 (0.8%) with family history and 12 (6.9%) with no family history. PAPM can identify the characteristics of a subject’s early stage of health behavior through the unawareness stage, and it can be seen from this study that there is a significant difference in the initial awareness of CRCS depending on the presence or absence of family history. Based on these results, having family history is an important mediating factor to move people in the unawareness and unengaged stages to the undecided-to-act stage in the CRCS behavior-change stage. 

Among the health beliefs, a perceived barrier is a negative aspect of certain health behaviors that make it difficult for people to take up appropriate health behaviors. In this study, it has been shown that the lower the behavioral change stage is, the higher the perceived barrier is. In particular, the perceived barrier of subjects in the unawareness stage was 4.10 times higher than that in the maintenance stage. This was consistent with the results of a study that reported that as the barrier factor decreased in the CRCS, the screening behavior-change phase increased toward the maintenance phase [17]. Furthermore, because it was reported that the awareness level of the need for screening is higher when the CRCS barrier factors are perceived to be fewer [7], an educational program is required to intervene with the lack of information about CRCS and inadequate awareness.

Next, the self-efficacy score was the highest in the decided-to-act stage and showed a decreasing trend as they moved to the unawareness stage. This is consistent with the result wherein self-efficacy increased as the gastric cancer screening behavioral-change stage increased in the maintenance direction [21]. It also supports the results of a previous study that found that self-efficacy was significantly higher for the subject group with CRCS experience compared to the group without it [7]. Comparing the characteristics of each stage of behavior change, the self-efficacy of the group that underwent the examination was higher than that of the group that did not. In particular, self-efficacy was significantly higher when subjects were in the acting stage than in the maintenance stage. As such, self-efficacy is an important determinant in the execution stage of performing health behavior [22,23]. Based on these research results, developing and applying a self-efficacy enhancement program to move the subjects from the undecided-to-act stage to the acting stage will be helpful. In addition, it is judged that active health behaviors can be maintained and improved by conducting a preventive campaign on the CRCS effect or improving self-efficacy through organizing meetings with people who have experienced screening.

Based on the foregoing, it is critical to select a target group considering the behavioral-change stage to establish a strategy for improving the CRCS rate. Gender, marital status, family history, private insurance, perceived barrier, and self-efficacy shown in the results of this study can be significant predictors for selecting the stage of CRCS behavior change. In addition, developing and implementing a CRCS education program will be a strategy to increase the examination of effectiveness of regular CRCS. 

While this study has been conducted to identify the stage of behavior change of CRCS recommended targets and the factors affecting each stage of the CRCS, there are a few limitations. First, since the data was collected from workers of an industrial complex, there is a limit to generalizing the results. Second, not all the variables affecting the change between stages suggested by PAPM have been considered. Third, there is no standardized module for classifying the CRCS behavior-change stages, so there is a limitation in that the change stages were arbitrarily applied by researchers. Fourth, there is a limitation in that predictive factors could not be analyzed considering all the changes in each stage because there were no subjects in the deciding-not-to act stage. Fifth, there is a limitation in comparing the characteristics of groups by stage due to the deviation of the number of subjects for each stage of CRCS behavior change. However, it is meaningful that this study presented basic data for developing nursing interventions that can increase the CRCS rate by identifying the stages of change in CRCS behavior and factors affecting each stage.

## 5. Conclusions

This study has been conducted to establish a strategy for improving the CRCS rate by applying PAPM to identify the stages of change in CRCS behavior and identify factors affecting each stage. As a result of examining the stages of behavior change for workers aged 50 years or more, the age at which the CRCS recommendation starts in South Korea, most of the subjects were found to be in the undecided-to-act stage. The influencing factors were gender, marital status, family history, private insurance, perceived barrier, and self-efficacy. In particular, education to reduce perceived barriers is required for subjects in the unawareness and unengaged stages. For those in the undecided-to-act stage, intervention focusing on social support and recommendation through their spouse is necessary. Education for enhancing self-efficacy should be applied to move from the undecided-to-act stage to the acting stage, and in the acting stage, a customized intervention plan for the family unit considering the family history should be developed to enhance the need for regular check-ups. Furthermore, effective information provision channels should be developed to provide differentiated education according to demographic and sociological characteristics that affect the screening behavior. Thus far, this study has presented an empirical basis for developing intervention programs that can induce a change in the perception and behavior of CRCS subjects.

## Figures and Tables

**Table 1:** Demographic characteristics according to CRCS behavior-change stages (n = 305).

Variable	Categories	Total	Stage 1 (n = 13)	Stage 2 (n = 22)	Stage 3 (n = 121)	Stage 5 (n = 26)	Stage 6 (n = 56)	Stage 7 (n = 67)	X^2^ or *F*	*p*
			M(SD) or n(%)							
Age (years)		53.2(4.8)	56.23(6.4)	53.77(2.7)	52.55(3.2)	52.65(3.1)	52.79(2.6)	54.36(5.1)	4.06	0.001 *
Gender	Male	151(49.5)	8(5.3)	12(7.9)	53(35.1)	2(1.3)	34(22.5)	42(27.8)	28.21	0.000 **
	Female	154(50.5)	5(3.2)	10(6.5)	68(44.2)	24(15.6)	22(14.3)	25(16.2)		
Education ^†^	High School≥	199(65.2)	5(11.4)	2(4.5)	17(38.6)	5(11.4)	6(13.6)	9(20.5)	-	0.241
	College≤	106(34.8)	8(3.1)	20(7.8)	102(39.7)	21(8.2)	49(19.1)	57(22.2)		
Marital status	Married	277(90.8)	8(2.9)	20(7.2)	111(40.1)	23(8.3)	52(18.8)	63(22.7)	18.04	0.003 *
	Single	28(9.2)	5(17.9)	2(7.1)	10(35.7)	3(10.7)	4(14.3)	4(14.3)		
Employment status ^†^	Fixed regulation	135(44.3)	5(3.7)	6(4.4)	47(34.8)	12(8.9)	32(23.7)	33(24.4)	-	0.111
	Non-regulation	114(37.3)	1(2.5)	2(5.0)	19(47.5)	3(7.5)	10(25.0)	5(12.5)		
	Other	56(18.4)	13(4.3)	22(7.2)	121(39.7)	26(8.5)	56(18.4)	67(22.2)		
Family history	Yes	132(43.3)	1(0.8)	12(9.1)	46(34.8)	13(9.8)	24(18.2)	36(27.3)	12.67	0.027 *
	No	173(56.7)	12(6.9)	10(5.8)	75(43.4)	13(7.5)	32(18.5)	31(17.9)		
Private insurance ^†^	Yes	247(81.0)	7(2.8)	13(5.3)	93(37.7)	25(10.1)	48(19.4)	61(24.7)	-	0.000 **
	No	58(19.0)	6(10.3)	9(15.5)	28(48.3)	1(1.7)	8(13.8)	6(10.3)		

^†^ Fisher’s exact test; * *p* < 0.05; ** *p* < 0.001.

**Table 2:** Differences in health beliefs and self-efficacy according to CRCS behavior-change stages (n = 305).

Variable	Categories	Stage 1 ^a^ (n = 13)	Stage 2 ^b^ (n = 22)	Stage 3 ^c^ (n = 121)	Stage 5 ^d^ (n = 26)	Stage 6 ^e^ (n = 56)	Stage 7 ^f^ (n = 67)	*F*	*p*	Scheffe Test
		M(SD)								
Health beliefs	Perceived sensitivity	2.63(0.60)	2.08(0.81)	2.34(0.77)	2.26(0.87)	2.30(0.94)	2.29(0.76)	0.82	0.537	-
	Perceived severity	2.78(0.74)	2.43(0.97)	2.86(0.75)	2.69(0.94)	2.76(0.80)	2.57(0.66)	1.87	0.098	-
	Perceived benefit	3.76(0.61)	3.97(0.46)	4.13(0.94)	4.03(0.51)	4.04(0.64)	4.15(0.51)	0.86	0.507	-
	Perceived barrier	3.29(0.44)	3.22(0.40)	3.06(0.56)	3.01(0.65)	2.97(0.59)	2.78(0.58)	3.62	0.003 *	-
Self-efficacy		3.45(0.37)	3.69(0.45)	3.84(0.56)	3.90(0.46)	3.97(0.53)	3.43(0.50)	6.16	0.000 **	a < d, e

a = unawareness; b = unengaged; c = undecided about acting; d = decided to act; e = acting, f = maintenance; * *p* < 0.05; ** *p* < 0.001.

**Table 3:** Influencing factors for each stage of CRCS behavior change (n = 305).

Characteristics	Categories	Stage 1 (n = 13)	Stage 2 (n = 22)	Stage 3 (n = 121)	Stage 5 (n = 26)	Stage 6 (n = 56)
		OR(95% CI)				
Age		0.53(0.08–3.45)	0.56(0.17–1.84)	1.37(0.68–2.75)	1.09(0.36–3.25)	1.14(0.51–2.59)
Gender	Male	0.16(0.01–3.95)	0.36(0.07–1.79)	0.22(0.07–0.65) *	0.04(0.00–0.60) *	0.23(0.06–0.91) *
Marriage	Yes	0.11(0.02–1.71)	0.45(0.13–1.54)	0.33(0.15–0.75) *	0.45(0.14–1.50)	0.58(0.22–1.57)
Family history	Yes	0.35(0.35–1.75)	1.51(0.52–4.38)	0.62(0.31–1.23)	1.49(0.50–4.47)	6.37(0.00–0.01) *
Private insurance	Yes	0.21(0.03–1.36)	0.18(0.02–1.32)	0.29(0.10–0.84) *	1.50(0.15–15.43)	0.49(0.14–1.73)
Perceived barrier		4.10(1.27–13.29) *	3.25(1.22–8.69) *	1.84(1.02–3.31) *	2.04(0.84–4.94)	1.64(0.84–3.23)
Self-efficacy		0.28(0.07–1.11)	0.49(0.23–1.03)	0.91(0.38–2.21)	2.28(0.69–7.52)	0.22(0.65–0.74) *

Reference group = maintenance stage (n = 67); dummy variables (Reference: gender = female; marriage = single; family history = no; private insurance = no); * *p* < 0.05.

## Data Availability

The data presented in this study are available on request from the corresponding author. The data are not publicly available due to restrictions, e.g., privacy or ethical.
